# Report of an Autopsy

**Published:** 1901-09

**Authors:** John Doe

**Affiliations:** Philadelphia


### REPORT OF AN AUTOPSY.

The following report was recently received:

PHILADELPHIA, August 7, 1901.

DR. (RICHARD ROE),

DEAR SIR: I made a close post mortem examination on the body of the horse which we removed from your hospital, and I found that the animal had inflammation in the Stomach and small Bowels and both Lungs had fallen apart. Liver was completely decomposed both Kidneys were very much effected.

Examined by

(JOHN DOE).

